# Metformina en el tratamiento de enfermedades dermatológicas: una revisión narrativa

**DOI:** 10.1016/j.aprim.2022.102354

**Published:** 2022-05-12

**Authors:** Juan Monte-Serrano, Pablo Villagrasa-Boli, Joana Cruañes-Monferrer, Patricia Arbués-Espinosa, Sara Martínez-Cisneros, Miguel Fernando García-Gil

**Affiliations:** a Servicio de Dermatología y Venereología, Hospital Clínico Universitario Lozano Blesa, Zaragoza, España; b Health Research Group GIIS100 of Health Research Institute IIS Aragón, Zaragoza, España; c Servicio de Dermatología y Venereología, Hospital Universitario Reina Sofía, Murcia, España; d Centro de Salud Delicias Norte, Sector Sanitario Zaragoza III, Zaragoza, España; e Servicio de Dermatología y Venereología, Hospital Clínico Universitario Miguel Servet, Zaragoza, España

**Keywords:** Metformina, Dermatología, Piel, Pleiotropismo farmacológico, Metformin, Dermatology, Skin, Pharmacological pleiotropism

## Abstract

**Objetivo:**

Revisar y discutir la evidencia actual del uso de la metformina como herramienta terapéutica en enfermedades cutáneas.

**Diseño:**

Artículo original. Investigación cualitativa. Revisión narrativa.

**Emplazamiento:**

Aragón y Murcia, España.

**Participantes:**

Médicos Internos Residentes de Dermatología Médico-Quirúrgica y Venereología y de Atención Primaria y Comunitaria.

**Métodos:**

Se ha realizado una revisión narrativa utilizando la base de datos bibliográfica PubMed con fecha de búsqueda el 27 de enero de 2022.

**Resultados:**

La metformina ha demostrado ser efectiva en el tratamiento de dermatosis inflamatorias tales como el acné, hidrosadenitis supurativa, psoriasis y dermatitis de contacto alérgica. También ha demostrado propiedades antitumorales frente al carcinoma basocelular, carcinoma espinocelular y melanoma. De forma adicional, se ha descrito efectos beneficiosos del tratamiento adyuvante con metformina en pacientes con carcinoma basocelular que reciben terapia fotodinámica. En pacientes con dermatosis relacionadas con endocrinopatías tales como el hirsutismo, la acantosis nigricans y los xantomas eruptivos, el tratamiento con metformina ha demostrado efectividad terapéutica. El tratamiento tópico con metformina ha demostrado ser eficaz en el tratamiento del melasma. Finalmente se ha propuesto como un fármaco con propiedades antienvejecimiento cutáneo y favorecedoras de la cicatrización. Para ninguna de las indicaciones previamente descritas se han objetivado efectos adversos graves.

**Conclusiones:**

La metformina es un tratamiento efectivo y seguro en el esquema terapéutico de dermatosis inflamatorias, neoplasias cutáneas, dermatosis relacionadas con endocrinopatías, melasma, envejecimiento cutáneo y cicatrización.

## Abstract (en)

**Objetive:**

To review and discuss the current evidence of the use of metformin as a therapeutic tool in frequent skin diseases.

**Design:**

Original article. Qualitative research. Narrative review.

**Location:**

Aragon and Murcia, Spain.

**Participants:**

Resident Physicians. Dermatology and Primary Health Care.

**Method:**

A narrative review has been carried out using the PubMed bibliographic database, being the search date the 27th of January of 2022.

**Results:**

Metformin has proven to be effective in the treatment of inflammatory skin diseases such as acne, hidradenitis suppurativa, psoriasis and allergic contact dermatitis. It has also shown antitumor properties regarding basal cell carcinoma, squamous cell carcinoma and melanoma. Additionally, beneficial effects of adjuvant treatment with metformin have been described in patients with basal cell carcinoma receiving photodynamic therapy. In patients with endocrinology-related dermatosis such as hirsutism, acanthosis nigricans and eruptive xanthomas, treatment with metformin has demonstrated therapeutic effectiveness. Topical treatment with metformin has also been useful in the treatment of melasma. Finally, it has been proposed as a drug with anti-aging and wound-healing promoting properties. Severe adverse effects have not been observed for any of the previously described indications, being this a well-tolerated treatment.

**Conclusions:**

Metformin is an effective and safe adjuvant in the therapeutic scheme of various inflammatory dermatoses, skin neoplasms, endocrinology-related dermatosis, melasma, skin aging and wound healing processes.

## Introducción

La metformina es una biguanida clásicamente utilizada como tratamiento de primera línea en el tratamiento de la diabetes mellitus tipo 2. En adultos, el tratamiento con metformina generalmente se inicia con dosis de 500 mg-850 mg dos veces al día con un incremento de 500 mg cada semana o 850 mg cada dos semanas pudiendo alcanzar una dosis diaria máxima de 2550 mg^1^.

Entre las contraindicaciones del empleo de la metformina destacan la hipersensibilidad al fármaco y pacientes con alto riesgo de acidosis láctica. Entre los pacientes con alto riesgo e acidosis láctica se incluyen: 1) insuficiencia renal moderada (aclaramiento de creatinina < 30 ml/min) o patologías con predisposición a alteración de la función renal tales como la deshidratación, infecciones graves o shock hipovolémico; 2) situaciones de acidosis metabólica aguda o crónica, incluida la cetoacidosis diabética con o sin coma diabético; 3) situaciones de hipoxemia, como insuficiencia cardíaca descompensada, insuficiencia respiratoria, infarto agudo de miocardio reciente o shock hipovolémico; 4) insuficiencia hepática, alcoholismo crónico o intoxicación etílica aguda.

Por lo que respecta a nuevas indicaciones terapéuticas de la metformina, en los últimos años se ha objetivado un interés creciente en el empleo de la metformina como una herramienta terapéutica fundamental para el abordaje del síndrome metabólico, ovario poliquístico, estados hiperandrogénicos y más recientemente en patologías dermatológicas^2^, ^3^.

En esta revisión narrativa se discute la evidencia actual de la metformina para el tratamiento de dermatosis inflamatorias, dermatosis relacionadas con endocrinopatías, neoplasias cutáneas, melasma, envejecimiento cutáneo y cicatrización.

## Material y métodos

Se ha realizado una revisión narrativa de los estudios disponibles en las bases de datos PubMed, en los cuales se ha empleado la metformina para el tratamiento de patologías dermatológicas.

Los términos Medical Subject Headings (MESH) incluidos fueron: «metformin», «dimethyl guanil guanidine», «dimethyl biguanide», «dose», «dermatology», «skin», «nail», «hair». La fecha de búsqueda fue el 27 de enero de 2022. Todos los artículos desde el inicio de la base de datos fueron incluidos en la búsqueda.

Solo se analizaron los estudios con resumen disponible que proporcionase información sobre el efecto terapéutico de la metformina en patología cutánea. Se incluyeron tanto trabajos *in vivo* como *in vitro*.

Con el objetivo de incluir más estudios (especialmente artículos pendientes de publicación, trabajos no publicados en PubMed o información de ficha técnica del fármaco), se realizaron búsquedas manuales en Google utilizando como palabras clave los MESH utilizados en PubMed.

## Resultados

### Dermatosis inflamatorias (tabla 1)

#### Acné

La metformina desempeña un papel en la patogénesis del acné, especialmente en aquellos de perfil hormonodependiente, puesto que su administración produce una disminución en los niveles del factor de crecimiento insulínico tipo 1 (IGF-1), molécula que a su vez estimula la síntesis de andrógenos y la conversión periférica de los mismos al activar la enzima 5-ɑ-reductasa, lo que regulará la producción de sebo en el folículo pilosebáceo^2^ ().

**Tabla 1:** Utilización de la metformina en dermatosis inflamatorias

Autor (año)	Tipo de trabajo	Resultados
Robinson et al. (2018)	Ensayo clínico	La metformina mejora el control del acné al utilizarse como adyuvante con peróxido de benzoilo tópico y doxiciclina oral
Theut et al. (2017)	Revisión narrativa/opinión de expertos	La metformina reduce la puntuación en la escala de gravedad Sartorius y la calidad de vida en pacientes con hidradenitis supurativa
Chang et al. (2020)	Revisión narrativa	La metformina ha mostrado ser beneficiosa en el tratamiento del acné, hidradenitis supurativa y psoriasis, y otras entidades inflamatorias como la dermatitis de contacto alérgica

La mejoría clínica del acné al introducir metformina en pacientes con y sin sobrepeso o resistencia insulínica, ha sido demostrada en numerosos ensayos clínicos y estudios de cohortes, con dosis de entre 500 y 2550 mg al día, asociada o no a otros tratamientos tópicos o sistémicos^2^, ^3^, ^4^.

#### Hidradenitis supurativa

La administración de metformina en pacientes con hidradenitis supurativa (HS) se ha relacionado con mejoras en la actividad de la enfermedad y calidad de vida de los mismos, reportándose mejor control de la enfermedad en pacientes diabéticos^2^, ^3^.

Además de la regulación de la secreción de sebo en el folículo, de manera análoga a lo ocurrido en el acné, la metformina posee actividad inflamatoria en los pacientes con HS a través de la inhibición del factor nuclear κB (NFκB), factor promotor de la síntesis de citocinas proinflamatorias^5^.

Su adecuada tolerancia lo convierte en una opción a tener en cuenta en la HS, al ser esta resistente a los tratamientos y una entidad en la que la modificación del estilo de vida, pérdida de peso y disminución de la resistencia insulínica son factores que mejoran el control de la misma^2^.

#### Psoriasis

La psoriasis es una enfermedad inflamatoria sistémica con expresión cutánea, en la que existe un ambiente molecular proinflamatorio mediado por diversas citocinas. Del mismo modo, los pacientes con psoriasis tienen mayor riesgo de desarrollar diabetes y síndrome metabólico^3^, ^6^.

La metformina activa la proteincinasa mediada por AMP (AMPK), la cual disminuye la actividad de las células dendríticas cutáneas, linfocitos T, macrófagos, células endoteliales y monocitos, todos ellos implicados en la patogénesis de la psoriasis^2^. Además, la metformina también reduce la síntesis de especies reactivas del oxígeno a nivel mitocondrial, lo que disminuye el estrés oxidativo y la respuesta inflamatoria local.

Hasta la fecha, se ha constatado que el beneficio clínico de la utilización de metformina en pacientes psoriásicos es constatado fundamentalmente en pacientes con diabetes o intolerancia a la glucosa^2^.

### Dermatosis relacionadas con endocrinopatías (tabla 2)

La metformina tiene efectos antiandrogénicos y de mejoría en la hiperinsulinemia que tienen un impacto positivo en diversas dermatosis como la acantosis nigricans, xantomas, el acné hormonal, el hirsutismo y trastornos de la pigmentación^2^, ^3^ ().

**Tabla 2:** Utilización de la metformina en dermatosis relacionadas con endocrinopatías

Autor (año)	Tipo de trabajo	Aspectos relevantes	Resultados	Conclusiones
Sung et al. (2020)	Revisión sistemática	Uso de la metformina en la acantosis nigricans Se incluyeron cinco casos clínicos, un ensayo clínico, dos cohortes prospectivas y una serie de casos	Metformina es eficaz para la AN, en el 72,2% de los pacientes empleando dosis entre 500 y 2000 mg al día. La metformina oral también fue eficaz en la AN junto con isotretinoína a dosis de 40-80 mg/día	El uso de metformina demostró mejoría clínica en el aspecto de las lesiones de AN en diversos tipos de estudio incluidos ensayos clínicos de forma aislada y combinada. Sin embargo, se requieren más estudios
Sung et al. (2020)	Revisión sistemática	Uso de la metformina en el hirsutismo. Se incluyeron ensayos clínicos y estudios de cohortes con diversas dosis y en combinación con otros tratamientos como espironolactona, el acetato de etinilestradiol-ciproterona (EE-CA), la N-acetilcisteína (NAC), el mioinositol y otros antidiabéticos	30 ensayos clínicos con resultados de eficacia variables. No presentaron mejoría en 4 ensayos clínicos y cinco estudios de cohortes	La eficacia del tratamiento del hirsutismo con metformina no es clara y se requiere de mayor evidencia
Patel-Nupur et al. (2018)	Revisión narrativa	Uso de la metformina en la acantosis nigricans. Se incluyeron 3 ensayos clínicos, una serie de casos y un caso clínico. Se analizó la eficacia de forma aislada como con tratamientos antidiabéticos combinados	Uso de forma aislada de metformina a dosis de 500 mg al día tres veces al día ha mostrado eficacia significativa en reducir las lesiones de AN. La combinación de metformina con rosiglitazona mostró una mejoría mínima de las lesiones de AN	La metformina puede ser eficaz en el tratamiento de la AN de forma asilada o en combinación con otros tratamientos
Streit-Helmbold et al. (2009)	Caso clínico	Uso de la metformina en los xantomas eruptivos. Hombre 65 años, con diagnóstico hiperlipidemia secundaria y de DM tipo II	Resolución de xantomas eruptivos con hiperpigmentación postinflamatoria tras 6 meses de tratamiento con metformina y bezafibrato	La metformina, gracias a la oxidación de los ácidos grasos y a la reducción de enzimas lipogénicas tiene efectos positivos en el tratamiento de los xantomas

Destacamos las siguientes patologías debido a la eficacia y mayores aplicaciones terapéuticas de la metformina.

#### Acantosis nigricans

La acantosis nigricans (AN) es una dermatosis relacionada con resistencia a la insulina, diabetes mellitus, obesidad, trastornos endocrinos o farmacológicos.

Los mecanismos patogénicos de la AN involucran la estimulación de receptores de la familia de las tirosina quinasa, provocando un defecto en la translocación del transportador de glucosa 4 (GLUT4) hacia la membrana plasmática de adipocitos y miocitos que potencian la actividad de los receptores del factor de crecimiento similar a la insulina (IGF-R) de queratinocitos y fibroblastos, lo que conlleva a la proliferación ambos tipos de células y la aparición de placas papilomatosas. También los niveles altos de insulina favorecen el aumento de la cantidad de IGF-1 libre circulante provocando el crecimiento y diferenciación de los queratinocitos^2^, ^7^.

El uso de la metformina para la hiperinsulinemia mediante la acción sobre el receptor GLUT4, mejora significativamente la AN. Se ha descrito la terapia combinada con otros tratamientos como tiazolidinedionas o glimepirida con metformina para potenciar su efecto en casos resistentes^2^, ^7^.

#### Hirsutismo

Se caracteriza por el crecimiento excesivo de vello terminal en áreas andrógeno-dependientes en la mujer. La prevalencia de hirsutismo es más elevada en pacientes con SOP, entre el 70% y el 80%, frente al 4 y el 11% en mujeres de la población general. Se piensa que la reducción de los niveles circulantes de insulina conlleva a una disminución de la concentración de andrógenos circulantes libres, por lo que la metformina puede tener efectos positivos en el hirsutismo^8^.

#### Xantomas eruptivos

Son depósitos de lípidos cutáneos generalmente asociadas a dislipidemia tipo I, IV, y V. La acción de la metformina activa la proteína quinasa activada por monofosfato de adenosina (AMPK) en los hepatocitos, reduciendo la actividad de la acetil-CoA carboxilasa, que conlleva a la oxidación de los ácidos grasos y reduce la expresión de enzimas lipogénicas. Se han descrito casos que han presentado mejoría para esta indicación^2^, ^9^.

### Neoplasias cutáneas (tabla 3)

La metformina presenta propiedades antitumorales para múltiples neoplasias, entre las que destacan el carcinoma basocelular, el carcinoma epidermoide cutáneo y el melanoma ().

**Tabla 3:** Utilización de la metformina en neoplasias cutáneas

Autor (año)	Tipo de trabajo	Resultados
Adalsteinsson et al. (2021)	Casos y controles	Pacientes tratados con metformina presentaban menor riesgo de desarrollar carcinoma basocelular (OR: 0,71)
Misitzis et al. (2021)	Cohortes	Pacientes tratados con metformina presentaban menor riesgo de desarrollar carcinoma basocelular (HR: 0,7) y carcinoma epidermoide (HR: 0,62).
Mascaraque et al. (2020)	Experimental in vivo e in vitro	La metformina podría resultar útil como tratamiento adyuvante en pacientes que reciben terapia fotodinámica para carcinoma basocelular

En cuanto a los mecanismos que podrían explicar estas propiedades quimiopreventivas, destaca el efecto activador de la metformina en la vía AMP quinasa, la cual, a su vez, inhibe la vía mTOR y estimula al gen supresor tumoral p53. Metformina también ha demostrado ejercer una función inhibidora directa de la vía Hedgehog, clave en el desarrollo del carcinoma basocelular. Asimismo, este fármaco inhibe la transición epitelio-mesenquimal y la actividad de las metaloproteasas en pacientes con melanoma, ejerciendo de este modo una función antimetastásica^2^.

#### Carcinoma basocelular

En el estudio de Adalsteinsson et al. se observó, en un grupo de 6880 pacientes islandeses, que aquellos tratados con metformina, independientemente de la dosis terapéutica, presentaban menor riesgo de desarrollar carcinoma basocelular (odss ratio: 0,71). No obstante, este trabajo presenta algunas limitaciones, ya que se trata de un estudio retrospectivo y además, no se tuvo en cuenta el fototipo de los pacientes ni su grado de exposición previa a radiación ultravioleta^10^.

#### Carcinoma epidermoide

Por otra parte, el estudio de cohortes de Misitzis et al. encontró asociación entre el uso de metformina y un menor riesgo para el desarrollo de carcinoma epidermoide cutáneo y de carcinoma basocelular. Concretamente, los pacientes en tratamiento con metformina presentaban un hazard ratio (HR) de 0,62 en cuanto a desarrollo de carcinoma epidermoide, en comparación con aquellos que no recibían tratamiento con metformina. El HR para el desarrollo de carcinoma basocelular fue de 0,70 para la misma comparación^11^.

#### Melanoma

Recientemente, se ha propuesto que la combinación de metformina y anti-VEGF podría ser útil en pacientes con melanoma BRAF y mutación V600E, resistentes a tratamiento con inhibidores BRAF. Esta hipótesis se basa en la observación in vitro del mayor crecimiento de células de melanoma BRAF mutado, tratadas con metformina, debido a la regulación al alza de VEGF. Además, cuando se añadían inhibidores de VEGF, el crecimiento de estas células era suprimido^12^.

#### Terapia fotodinámica

Asimismo, este fármaco también ha demostrado utilidad como tratamiento adyuvante en pacientes que reciben terapia fotodinámica para carcinoma basocelular. Los autores demostraron que aquellas células cancerígenas resistentes a terapia fotodinámica, en pacientes con carcinoma basocelular, mostraban un metabolismo predominante de glucólisis aerobia. El tratamiento con metformina arrestaba a estas células en fase G0/G1 y producía una reducción en la producción de lactato por parte de las mismas. De hecho, la adición de metformina a MAL-PDT mejoraba su capacidad citotóxica sobre células resistentes a terapia fotodinámica^13^.

### Hiperpigmentaciones cutáneas, fotoenvejecimiento y cicatrización (tabla 4)

#### Hiperpigmentaciones cutáneas

Recientemente, se ha estudiado el papel de la metformina tópica, formulada al 30% en una solución de alcohol y propilenglicol, en el tratamiento de trastornos que cursen con hiperpigmentación cutánea, demostrando esta su efectividad en estudios de laboratorio en modelos animales^2^. Sin embargo, estos beneficios se han constatado únicamente con su aplicación tópica, sin existir mejoría al administrar el fármaco por vía sistémica. La metformina reduce la expresión de 3 proteínas melanogénicas: la tirosinasa, la proteína relacionada con tirosina (TRP) 1 y la TRP-2, lo que disminuye la producción local de melanina y con ello la hiperpigmentación^3^ ().

**Tabla 4:** Utilización de la metformina en hiperpigmentaciones cutáneas, fotoenvejecimiento y cicatrización

Autor (año)	Tipo de trabajo	Resultados
Aditya et al. (2016)	Revisión sistemática	La metformina tópica es efectiva en el tratamiento de hiperpigmentaciones cutáneas
Sung et al. (2020)	Revisión sistemática	La metformina actúa sobre 3 proteínas melanogénicas: tirosinasa, proteína relacionada con tirosina (TRP)-1 y TRP- 2 provocando una reducción en su expresión
Xiao et at. (2021)	Ensayo clínico	La metformina posee funciones antiinflamatorias y citoprotectoras en queratinocitos expuestos a UVB y ratones irradiados con UVB
Low et al. (2021)	Revisión narrativa	La senescencia celular es potencial causa de fotoenvejecimiento
Pils, et al. (2021)	Ensayo clínico	La metformina es efectiva como fármaco senolítico
Zhao et al. (2017)	Ensayo clínico	La metformina tópica se postula como un agente regenerador óptimo y prometedor en el tratamiento cutáneo de las cicatrices

#### Fotoenvejecimiento

Existen varios factores relacionados con el fotoenvejecimiento. Por un lado, la inflamación excesiva y la muerte celular inducida por los rayos ultravioleta (UV) provocan envejecimiento en la piel. En estudios recientes con modelo animal, se han evidenciado las funciones antiinflamatorias y citoprotectoras de la metformina en queratinocitos expuestos a UVB y ratones irradiados con UVB^14^. Por otro lado, la senescencia celular también ha demostrado en varios estudios ser causa potencial de fotoenvejecimiento^15^. La fisiopatología es compleja ya que están implicados varios tipos de células diferentes incluidos los fibroblastos, los queratinocitos y los melanocitos. Se ha propuesto a la metformina como un fármaco senolítico, cuya función es eliminar específicamente las células senescentes y reparar el daño producido por la radiación UV^16^.

#### Cicatrización

Las heridas cutáneas se encuentran entre las lesiones de tejidos blandos más comunes y son particularmente difíciles de curar con el envejecimiento de la piel. Existe evidencia de que la restricción calórica favorece la longevidad. Se han descubierto efectos rejuvenecedores de la piel utilizando fármacos miméticos de la restricción calórica, especialmente metformina, resveratrol y rapamicina. Se ha observado que la administración tópica crónica de metformina y resveratrol acelera la cicatrización de heridas debido a su efecto reparador de las alteraciones en la epidermis, los folículos pilosos y favorecedor de la síntesis de colágeno en roedores. Ambos fármacos, aplicados localmente, mejoran la vascularización en el lecho de las heridas. En la piel envejecida se observa una inhibición en la vía de la proteína quinasa activada por monofosfato de adenosina (AMPK), lo que se traduce en alteraciones en la vascularización y una reducción en la capacidad de cicatrización. El tratamiento local con metformina previene la supresión de AMPK relacionada con la edad^17^.

## Conclusiones y limitaciones

La metformina ha demostrado su utilidad en el tratamiento de dermatosis inflamatorias como el acné, psoriasis e hidradenitis, siendo mayor su efecto beneficioso en pacientes diabéticos o con síndrome metabólico. El mecanismo por el que dicha molécula interviene sobre la patogénesis de la HS y la psoriasis no es del todo conocido y serían necesarios ensayos clínicos aleatorizados.

La metformina tiene diversas aplicaciones en dermatosis relacionadas con endocrinopatías por sus efectos antiandrogénicos y antidiabéticos especialmente en la acantosis nigricans y el hirsutismo. Los estudios realizados hasta la fecha han obtenido resultados variables y muchos de ellos son casos clínicos por lo que se requiere de más ensayos para valorar y cuantificar la eficacia y seguridad.

La metformina presenta potencial actividad antitumoral en pacientes con carcinoma basocelular, carcinoma epidermoide cutáneo y melanoma. Algunos de los estudios presentados son de carácter observacional y retrospectivo. No existen ensayos clínicos en los que se haya demostrado este efecto antitumoral.

La metformina se presenta como un fármaco seguro y efectivo como terapia adyuvante en hiperpigmentaciones cutáneas, fotoenvejecimiento y cicatrización. Aunque existen trabajos en modelo animal que demuestran el efecto beneficioso de la metformina tópica en hiperpigmentaciones cutáneas, fotoenvejecimiento y cicatrización serán necesarios más trabajos en piel humana para comprender mejor estos mecanismos.

## Responsabilidades éticas

No se han utilizado datos de pacientes para la elaboración del presente trabajo. Los resultados del estudio forman parte del proyecto de tesis doctoral del Dr. Juan Monte Serrano, para el cual se cuenta con un dictamen favorable del Comité de Ética de Investigación Asistencial de Aragón (CEICA), encontrándose registrado en el Acta N.° 11/2021 de dicha institución.

## Autoría/colaboradores

Todos los autores han contribuido de forma sustancial en la elaboración del manuscrito.

## Financiación

Este trabajo no ha recibido ningún tipo de financiación.

## Conflicto de intereses

Los autores declaran no tener ningún conflicto de intereses.

**Lo conocido sobre el tema**

• La metformina es un fármaco de primera línea en el tratamiento de la diabetes mellitus tipo 2.
• La metformina ha demostrado efectos terapéuticos beneficiosos en el abordaje del síndrome metabólico, ovario poliquístico y otros estados hiperandrogénicos.
• Recientemente se han descrito propiedades terapéuticas de la metformina para distintas patologías dermatológicas.

**Qué aporta este estudio**

• Revisión de la evidencia actual del uso de la metformina como herramienta terapéutica en dermatosis inflamatorias y dermatosis relacionadas con endocrinopatías.
• Revisión de la evidencia actual del uso de la metformina como herramienta terapéutica en neoplasias cutáneas.
• Revisión de la evidencia actual del uso de la metformina como herramienta terapéutica en el melasma, envejecimiento cutáneo y cicatrización.
